# The Effect of Mitochondria on *Ganoderma lucidum* Growth and Bioactive Components Based on Transcriptomics

**DOI:** 10.3390/jof8111182

**Published:** 2022-11-09

**Authors:** Liyun Ye, Xiaofang He, Congbao Su, Haiying Feng, Guoliang Meng, Bingzhi Chen, Xiaoping Wu

**Affiliations:** 1 College of Life Sciences, Fujian Agriculture and Forestry University, Fuzhou 350002, China; 2 College of Food Sciences, Fujian Agriculture and Forestry University, Fuzhou 350002, China; 3 Key Laboratory of Subtropical Characteristic Fruits, Vegetables and Edible Fungi Processing (Co-Construction by Ministry and Province), Ministry of Agriculture and Rural Affairs, Fuzhou 350002, China

**Keywords:** isonuclear alloplasmic, yield, polysaccharide, triterpenoid, transcriptome

## Abstract

Mitochondria are the power source of living cells and implicated in the oxidative metabolism. However, the effect of mitochondria on breeding is usually ignored in conventional research. In this study, the effect of mitochondria on *Ganoderma lucidum* morphology, yield, and main primary bioactive components was analyzed via structuring and comparing isonuclear alloplasmic strains. The crucial biological pathways were then explored based on the transcriptome. The results showed that isonuclear alloplasmic exhibited difference in mycelial growth rate in potato dextrose agar medium (PDA), basidiospore yield, and polysaccharide and triterpenoid content. Otherwise, mitochondria did not change colony and fruit body morphology, mushroom yield, or mycelial growth rate in solid-state fermentation cultivation material. The transcriptome data of two significant isonuclear alloplasmic strains S1 and S5 revealed that the involvement of differentially expressed genes (DEGs) was mainly in pentose and glucuronate interconversions, starch and sucrose metabolism, and steroid biosynthesis. The result was further confirmed by the other isonuclear alloplasmic strains. The above results further proved that mitochondria could affect the active components of *G. lucidum*. Our results provide information which will contribute to understanding of mitochondria and will be helpful for breeding improved varieties.

## 1. Introduction

Mitochondria are organelles of eukaryotic cells that provide the platform for efficient energy metabolism and participate in the Krebs cycle to form ATP. Moreover, many studies showed that mitochondria are related to apoptosis, senescence, virulence, and drug resistance [1,2,3]. Elucidating the structure and function of the genome is important to understand species. Fungi mitochondria are semi-autonomous organelles and carry their own mitochondrial genomes (mtDNAs), most of which are closed circular super-helical DNA molecules. Different fungi mtDNAs usually present variability in size due to intergenic spacers, duplications, proliferation of repeats, and insertions of plasmid components or other elements [4,5,6]. With the continuous research and the development of new techniques, many edible fungi mtDNAs have been analyzed and annotated, such as *Agaricus bisporus*, *Agrocybe aegerita*, *Grifola frondose*, and *Sparassis crispa* [7,8,9,10]. In term of *G. lucidum*, Li et al. [11] assembled and analyzed the mtDNA of *G. lucidum* and clarified that the protein-coding genes were expressed higher in mycelia or primordial stages compared with those in the fruiting bodies. Li et al. [12] compared features, evolution, and phylogeny of five *Ganoderma* species mtDNAs. These results laid a foundation for the further study of mitochondrial function of *G. lucidum.*

Although most of the genetic information is stored in the nucleus, mitochondria also contain their own mtDNAs and mechanisms of replication and transcription. Mitochondria will not only directly affect the changes of organisms, but interact with nuclear genes to play a regulatory role. Presently, several studies clarified that mitochondrial and nuclear interactions, nuclear mitochondrial compatibility, and co-adaptation played an important role in fungal evolution and adaptation [1,13]. Moon et al. [14] found that the change of mitochondrial genetic background in mice would lead to significant changes in metabolites, obesity, and nuclear gene expression. Latorre-Pellicer et al. [15] study indicated that mtDNA influences mitochondrial proteostasis and reactive oxygen species (ROS) generation, insulin signaling, obesity, and ageing parameters, resulting in profound differences in health longevity.

*G. lucidum* is a medicinal fungus belonging to the family Polyporaceae in Basidiomycete. Modern pharmacologic studies have revealed that *G. lucidum* contains a variety of abundant pharmacological and biological active substances, such as polysaccharides, nucleosides, triterpenoids, peptides, sterols, protein, and alkaloids [16,17,18]. Therein, triterpenoids and polysaccharides are considered as the primary bioactive components that contribute to its medicinal properties. *G. lucidum* attracted various research interests due to its auxiliary function on anti-inflammatory, antitumor (both in vitro and in vivo), hepatoprotection, and some other activities [19,20,21,22,23]. Therefore, improvement of bioactive component production efficiency in *G. lucidum* has become a research focus in recent years.

Isonuclear alloplasmic, first named from isonuclear-alloplasmic male sterile lines in plant, indicated strains with the same nucleus and different mitochondrial genomes. The objective of the study reported here was to explore the effect of mitochondria on *G. lucidum* morphology, yield, and main primary bioactive components via comparing isonuclear alloplasmic strains. More than that, the mechanism of difference among test strains was analyzed based on the transcriptome. This information will be helpful for the further understanding of mitochondria function and provide a reference for the screening of excellent strains.

## 2. Materials and Methods

### 2.1. Strains

*G. lucidum* monokaryon strain M119-M71, M119-M54, MWBL, MTL, MSL4, and MSL10 were obtained from dikaryon strains 119, WBL, TL, SL4, and SL10 via protoplast method. All strains are currently preserved at the Mycological Research Center of Fujian Agriculture and Forestry University, Fujian, China. The mtDNAs of the *G. lingzhi* strains were submitted to GenBank and the accession numbers are shown as follows: M119-M71/M119-54: MT843216; MWBL: MT765268; MTL: MT843209; MSL4: MT843211; and MSL10: MT843212.

### 2.2. Isonuclear Alloplasmic Strains Obtention

**Figure 1:**
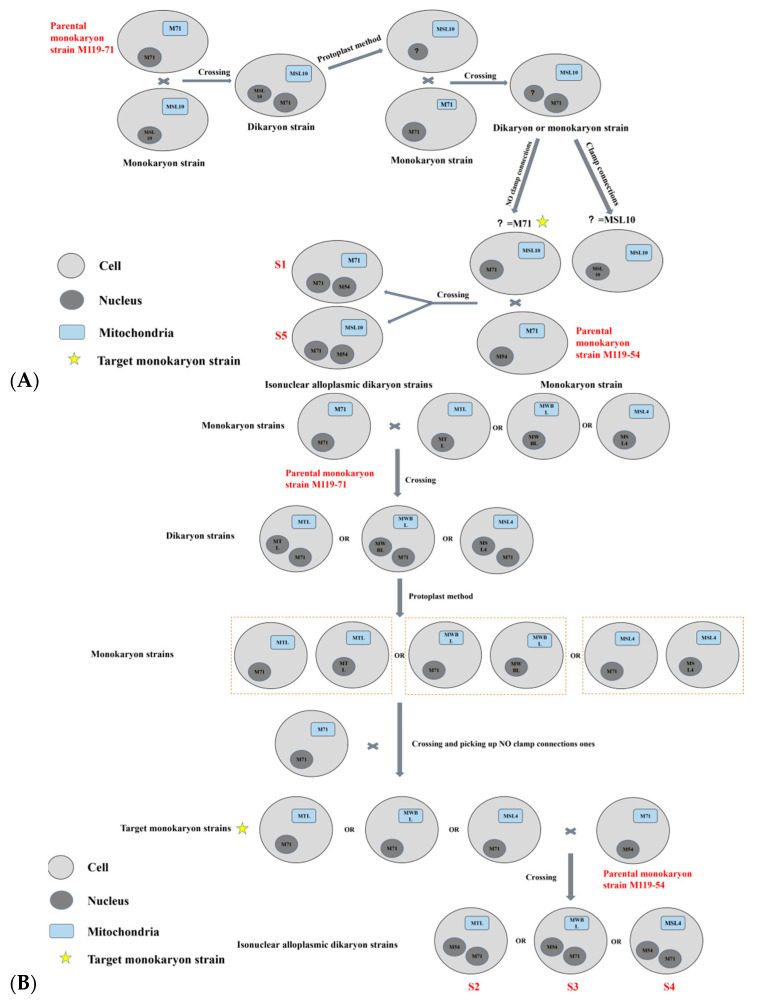
The process of constructing isonuclear alloplasmic. (**A**) The constructed strains S1 and S5. (**B**) The constructed strains S2, S3, and S4.

As shown in Figure 1, monokaryon strain M119-M71 and MSL10 were crossed on the plate. On the near MSL10 side, a dikaryon strain with M71-MSL10 nuclei and MSL10 mitochondria was obtained [24]. Next, protoplast method was used to produce a monokaryon strain with M119 or MSL10 nucleus and MSL10 mitochondria. The newly obtained monokaryon strain was backcrossed with M119-M71 and the type of nucleus was preliminary judged by clamp connection. Picked-up strains without clamp connection as the target monokaryon strain, for which nucleus was M119-M71-type and mitochondria was MSL10-type, were crossed with monokaryon strain M119-M54 to obtain dikaryon strain S1 and S5. S1 and S5 contained the same type nucleus but different mitochondria, M71 and MSL10, separately. Similarly, monokaryon strain M119-M71 was crossed with monokaryon strain MTL, MWBL, and MSL4 to obtain the target monokaryon strain with M119-M71-type nucleus and different mitochondria. Dikaryon strains S2 (MTL-type mitochondria), S3 (MWBL-type mitochondria), and S4 (MSL4-type mitochondria) were obtained by crossing target monokaryon strain with strain M119-M54. Strain S1, S2, S3, S4, and S5 were tested as isonuclear alloplasmic strains, which needed to be verified.

### 2.3. Isonuclear Alloplasmic Strains’ Nuclei DNA and mtDNA Proof

The whole genome of strains S1–S5 were sequenced using a HiSeq 2500 Illumina sequencer at Novogene Bioinformatics Institute (Shanghai, China). Obtained clean data were assembled into contigs by software SPAdes-3.7.1 with a Kmer 127. Relative mitochondrial proteins NCBI website was downloaded and mitochondria-related contigs were picked out from the assemblies by program Blastx. Mitochondria-related contigs were linked and then assembled into an intact circular molecule using the pair-end relationship of reads [24]. The mtDNAs of strains S1–S5 were compared using the Clustal W program (http://www.genome.jp/tools/clustalw/, accessed on: 16 October 2021). Nonidentical regions in the mtDNAs sequences were identified and four pairs of primers were designed to amplify the mtDNAs of *G. lucidum* strains S1–S5 using online tool Primer-BLAST (https://www.ncbi.nlm.nih.gov/tools/primer-blast/, accessed on: 16 October 2021) (Table S1). Similarly, primers of different nuclear specific fragments were designed among those *G. lucidum* strains (Table S1).

### 2.4. Biological Characteristics Observation

Mycelia were grown routinely at 25 °C on potato dextrose agar medium (PDA). Mycelial growth rate was determined by the ratio of mycelial extension distance to time. The cultivation material used for solid-state fermentation was composed of 39% sawdust, 39% cottonseed shell, 17% bran, 3% corn flour, 1% gypsum, and 1% light calcium carbonate, with 65% moisture content. The mixed substrate (1000 g) was transferred into a polypropylene bag (18 cm in width × 36 cm in length). Isonuclear alloplasmic strains’ fruit body and basidiospore yield were determined.

### 2.5. Total Polysaccharide and Triterpenoid Detection

The dried mycelia were ground into a fine powder. Total polysaccharide and triterpenoid extraction and detection was performed according to existing reports [25,26]. Polysaccharide was extracted by water and detected by the phenol-sulfuric acid method. The extraction method of triterpenoid was alcohol extraction and the detection method was vanillin–glacial acetic acid.

### 2.6. Total RNA Isolation and Transcriptome Analysis

Total RNA was extracted using a Total RNA Extraction Kit (Omega Bio-Tek, Guangzhou, China) according to the manufacturer’s instructions, and then the concentration and quality of RNA were detected. High-quality RNA was sent to Novogene (Beijing, China) for RNA-seq libraries preparation and sequenced using an Illumina HiSeq. Low-quality sequences and contamination were removed using FASTX toolkit (http://hannonlab.cshl.edu/fastx_toolkit, accessed on: 10 February 2022) to obtain clean reads, followed by mapping using HISAT2 [27]. The gene expression levels for each sample were expressed according to the fragments per kilobase per million (FPKM). |log_2_(Fold Change)| > 1 and q value < 0.05 were used as criteria of significant difference to identify differentially expressed genes (DEGs) [28]. For DEGs, GOseq software was used for gene ontology (GO) enrichment analysis based on Wallenius noncentral hypergeometric distribution [29], and Kyoto Encyclopedia of Genes and Genomes (KEGG) enrichment analysis was performed online according to KEGG database (https://www.kegg.jp/, accessed on: 18 May 2022).

### 2.7. Statistical Analysis

All data are expressed as the mean ± standard deviation of three independent experiments. Data were subjected to analysis of variance using the Student’s *t*-test, and the mean values indicating statistical significance were compared by Duncan’s multiple-range test using SPSS 20.0 (SPSS Inc., Chicago, IL, USA). *p* < 0.05 denoted statistical significance.

## 3. Results

### 3.1. Isonuclear Alloplasmic Proof

**Figure 2:**
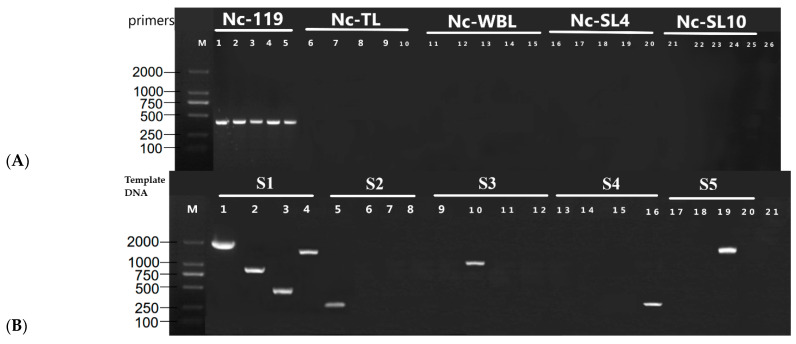
The PCR amplification of isonuclear alloplasmic strain S1–S5 nuclei and mitochondrion DNA. (**A**) The PCR amplification of strain S1–S5 nuclear genome. M indicates the 2000 bp DNA marker; the primers of lanes 1–5, 6–10, 11–15, 16–20, and 21–25 were Nc-119, Nc-TL, Nc-WBL, Nc-SL4, and Nc-SL10, separately; the template DNA of lane 1, 6, 11, 16, and 21 was S1; the template DNA of lane 2, 7, 12, 17, and 22 was S2; the template DNA of lane 3, 8, 13, 18, and 23 was S3; the template DNA of lane 4, 9, 14, 19, and 24 was S4; the template DNA of lane 5, 10, 15, 20, and 25 was S5; lane 26 is negative control with double-distilled water instead of DNA. (**B**) The PCR amplification of strain S1–S5 mtDNA. M indicates the 2000 bp DNA marker; the template DNA of lane 1–4, 5–8, 9–12, 13–16, and 17–20 were S1–S5, separately; the primers of 1, 5, 9, 13, and 17 were primer 6; the primers of 2, 6, 10, 14, and 18 were primer 7; the primers of 3, 7, 11, 15, and 19 were primer 8; the primers of 4, 8, 12, 16, and 20 were primer 9; lane 21 is negative control with double-distilled water instead of DNA.

According to different fragments between nuclear genomes, five pairs of primers were designed and the amplificated results showed that all strains only present the same bright band by the primer Nc-119, which was designed to distinguish M119 nuclear genome (Figure 2A). This means that all tested isonuclear alloplasmic strains contained the same style nuclear genomes M119. The amplification results of the mtDNAs of the constructed strains are shown in Figure 2B. Strain S1 amplified four different bands as expected. It indicated that S1 mtDNA was M119-style. Strain S2 only had a band by primer designed for MTL style, explaining that S2 mtDNA was MTL-style. Similarly, it was confirmed that strains S3, S4, and S5 contained MWBL, MSL4, and MSL10 mtDNA style, separately. This further concluded that strains S1–S5 were isonuclear alloplasmic strains containing the same style nucleus but different mitochondrion.

### 3.2. Mycelia and Fruit Body Morphology Observation

**Figure 3:**
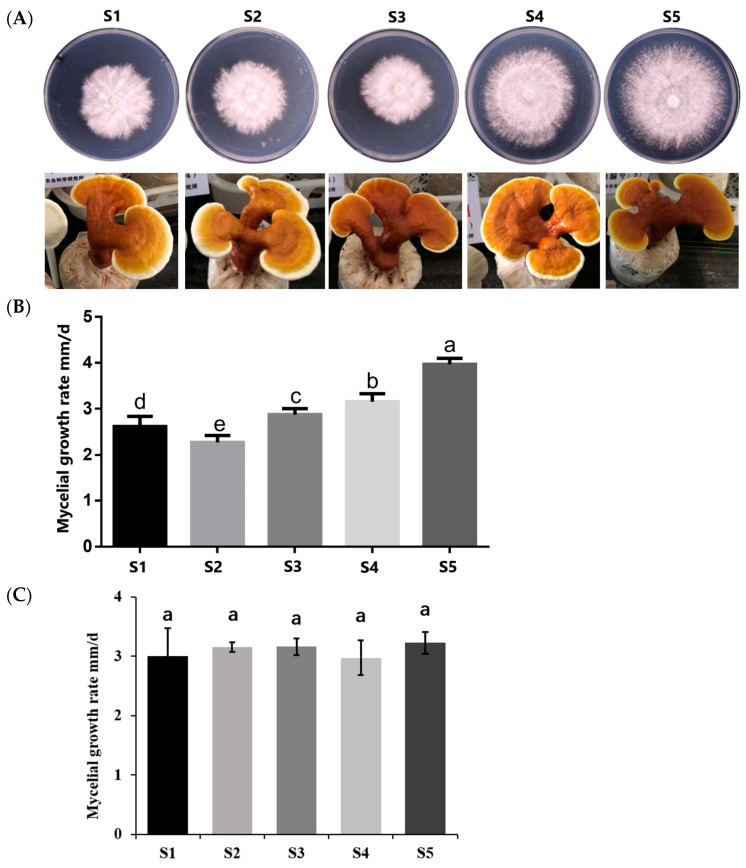
Biological characteristics of five isonuclear alloplasmic strains. (**A**) Colony and fruit body morphology. (**B**) Mycelial growth rate in PDA. Different lowercase a, b, c, and d indicate the significant difference among strains at the significance level of 0.05 (*p* < 0.05). (**C**) Mycelial growth rate in solid-state fermentation. The same lowercase a indicates no significant difference among strains at the significance level of 0.05 (*p* > 0.05).

The colony morphology and growth rate of five isonuclear alloplasmic strains were studied on PDA culture medium. The morphology of colonies was similar, with dense hyphae in the center and regular edges (Figure 3A). Conversely, the tested strains showed different mycelial growth rate. The fastest growth rate strain S5 was 4.04 mm/day, which was 1.81 times that of the slowest strain S2. Furthermore, the growth rate among the five strains was significantly different at the level of 0.05 (Figure 3A,B). Interestingly, their growth in solid-state fermentation cultivation material is similar, about 3.095 ± 0.125 mm/day (Figure 3B). The fruiting bodies of the five strains were difficult to distinguish. They all had umbrella shape and light reddish-brown pileus with a few rings (Figure 3A). It is concluded that different mitochondria affected the mycelial growth rate in PDA but did not affect the mycelial growth rate in solid-state fermentation cultivation material, colony, and fruit body morphology.

### 3.3. Comparative Analysis of Yield and Polysaccharides and Triterpenoid Content

The fruit body yield, basidiospore yield, and polysaccharides and triterpenoid content of isonuclear alloplasmic strains S1–S5 were measured and compared. As shown in Table 1, there was no statistical difference (*p* > 0.05) in mushroom yield among five tested strains. However, isonuclear alloplasmic strains showed significant difference in basidiospore yield (*p* < 0.05). The basidiospore yield of strain S1 was about 2.54 times higher than that in strain S5 (5.55 ± 0.99 g/bag). In addition, the content of polysaccharides was different among these tested strains. Polysaccharide content of strain S1 was highest, which was 20.17% higher than that in strain S3 (25.93 ± 0.85 mg/g). In contrast, the content of triterpenoid of strain S1 was significantly lower than that in the other four strains (*p* < 0.05). Hence, one can see that different mitochondria did not affect mushroom yield, but they affect the basidiospore yield and polysaccharide and triterpenoid content.

### 3.4. Assessment and Analysis of Transcriptome

**Figure 4:**
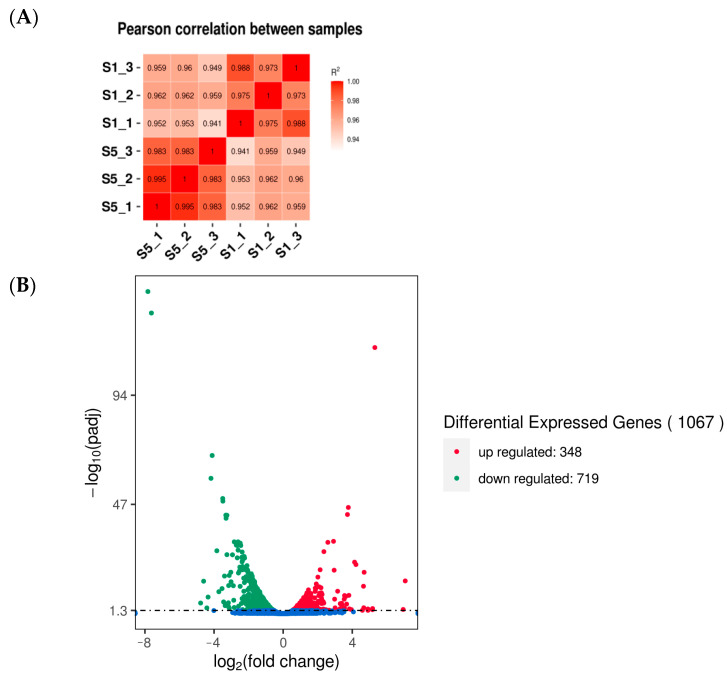
Correlation and DEGs analysis between strain S1 and S5. (**A**) Pearson correlation analysis between samples of strain S1 and S5. “_” indicated three biological duplications in each group. (**B**) DEGs between strain S1 and S5.

Compared to others strains, strains S1 and S5 exhibited significant difference in mycelial growth rate in PDA, basidiospore yield, and polysaccharide and triterpenoid content. In order to further understand the effects of different mitochondria on the growth, the complete transcriptome of *G. lucidum* isonuclear alloplasmic strain S1 and S5 with the same nuclear DNA and different mtDNAs was studied to explore the main mechanism of differences forming. Pearson’s correlation analysis between samples was performed and the result showed that R^2^ > 0.941, indicating that the biological replicates were reliable and transcriptome assembly was robust and available (Figure 4A, Table S2). The transcriptome analysis revealed a total of 1067 DEGs between strain S1 and S5. Compared to strain S1, 348 genes were upregulated and 719 genes were downregulated in strain S5 (Figure 4B).

### 3.5. Enrichment Analysis of DEGs

**Figure 5:**
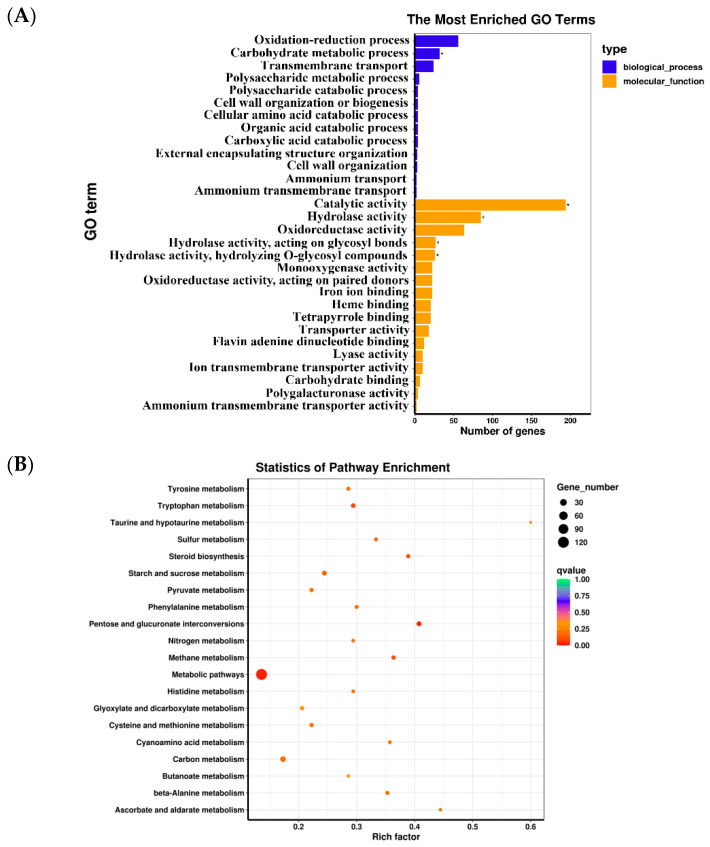
Enrichment analysis of DEGs. (**A**) GO enrichment of DEGs. (**B**) Bubble plot of KEGG enrichment of DEGs.

GO and KEGG enrichment analyses of DEGs were performed to explore the main difference formation mechanism between isonuclear alloplasmic strains. The result of GO enrichment analysis is shown in Figure 5A. In the biological process category, DEGs were mainly enriched in the oxidation–reduction process, carbohydrate metabolic process, and transmembrane transport. In the molecular function category, DEGs were mainly enriched in catalytic activity, hydrolase activity, and oxidoreductase activity. Gene enrichment analysis of DEGs based on the KEGG database revealed that these genes were mainly involved in pentose and glucuronate interconversions, starch and sucrose metabolism, and steroid biosynthesis, which were closely related to the active components of *G. lucidum* (Figure 5B).

### 3.6. Analysis of Bioactive-Component-Related Biological Pathways

**Figure 6:**
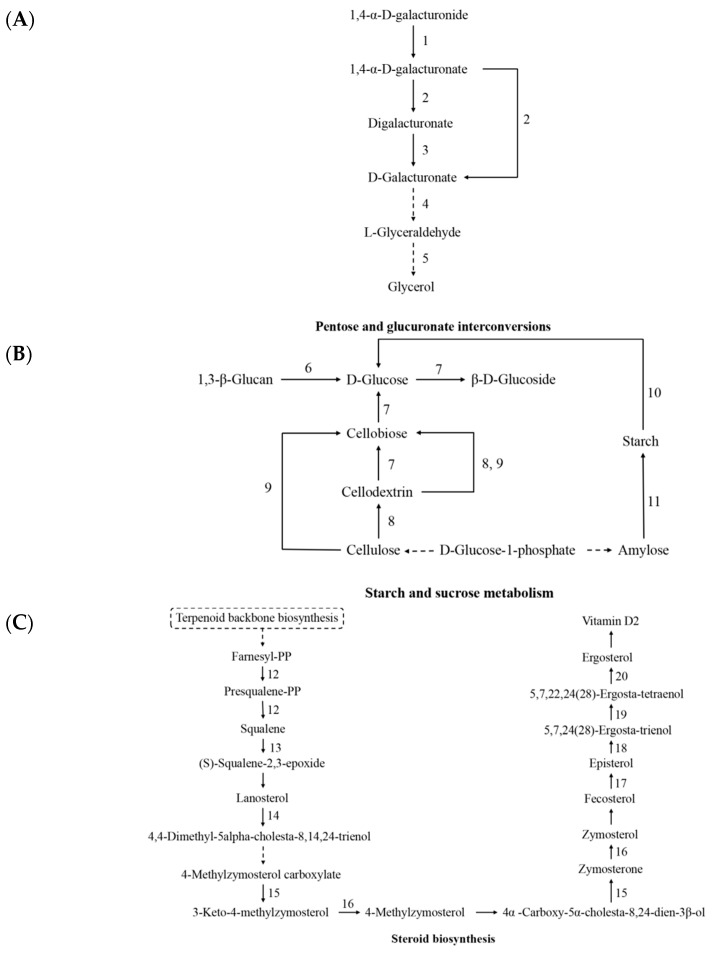

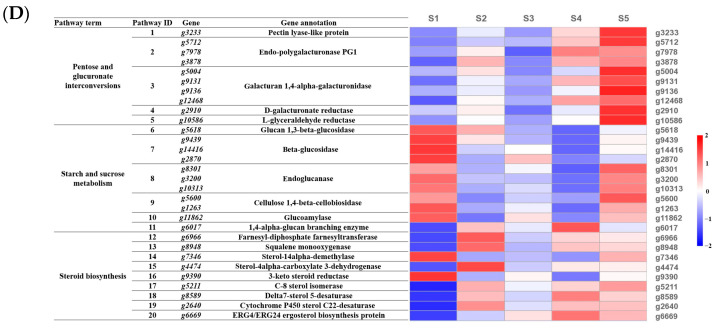
Polysaccharide- and triterpenoid-related pathways of isonuclear alloplasmic based on transcriptome analysis. Dashed lines indicate indirect connections or unknown reactions. The number along the arrows indicate pathway IDs in (**C**). (**A**) Pentose and glucuronate interconversions pathway. (**B**) Starch and sucrose metabolism pathway. (**C**) Steroid biosynthesis pathway. (**D**) Changes in expression of genes involved in pathways. Red to blue color indicated the highest to lowest relative expression level.

From the above results, it was concluded that mitochondria affected the content of polysaccharide and triterpenoid, which may relate to starch and sucrose metabolism, steroid biosynthesis, and pentose and glucuronate interconversions, according to the enrichment analysis of DEGs of strain S1 and S5 (Figure 5B). In these pathways, the expression of most genes differed not only in strain S1 and S5, but also differed in the other three isonuclear alloplasmic strains. In the pathway of pentose and glucuronate interconversions, most genes showed high expression in strain S5 and low expression in strain S1 and S3 (Figure 6A,D). In the starch and sucrose metabolism pathway, gene expression in strain S1 was significantly higher than that in strain S4, except for 1,4-alpha-glucan branching enzyme gene (*g6017*) (Figure 6B,D). In the pathway of steroid biosynthesis, except for sterol 14-alpha-demethylase gene (*g7346*, Figure 6D) and 3-keto steroid reductase gene (*g9390*, Figure 6D), genes showed a low expression level in strain S1 and high expression level in strain S4 (Figure 6C,D). These results confirmed the important role of the pathway of pentose and glucuronate interconversions, starch and sucrose metabolism, and steroid biosynthesis in the content of polysaccharide and triterpenoid.

## 4. Discussion

In this study, five isonuclear alloplasmic strains S1–S5 of *G. lucidum* were built and proved in order to explore the effect of mitochondria on *G. lucidum* morphology, yield, and main primary bioactive components. The results showed that different mitochondria lead to different mycelial growth rate in PDA, basidiospore yield, and the content of polysaccharide and triterpenoid.

Polysaccharides and triterpenoid, as the main active components, directly reflect the quality of *G. lucidum*. For a long time, many workers have tried many different approaches to improve the production of polysaccharides and triterpenoid. Wei et al. [30] obtained the highest intracellular polysaccharide production by a sucrose fed-batch strategy. Tang et al. [31] reported that added concentration of 5 mM Cu^2+^ on the culture of day 4 led to higher production of polysaccharides. The work of Sun et al. [32] demonstrated that promoting sporulation effectively improves GA production in *Ganoderma* species. Ma et al. [33] screened mutated high production of polysaccharide strains from plasma mutagenesis. It is worth noting that the effect of mitochondria on active components is usually ignored in conventional research. However, the result of this paper showed that different mitochondria led to different content of polysaccharides and triterpenoid in *G. lucidum*. To our knowledge, no published studies discuss the relation of mitochondria and active components. Shi et al. [34] found that the change in ROS would affect the production of *Ganoderma* acid. ROS were produced during energy metabolism in mitochondria [35,36]. Here, we suspect that the different result of isonuclear alloplasmic may be due to the difference in respiratory transmission chain, which then affects ROS, resulting in the difference in triterpenoid content. The mechanism of mitochondria’s effect was further investigated, analyzed based on transcriptome. In this paper, strains S1 and S5 were representative isonuclear alloplasmic because of the significant difference among mycelial growth rate in PDA, basidiospore yield, and polysaccharide and triterpenoid content. Their complete transcriptomes were analyzed. DEGs between strains S1 and S5 were enriched in biological process and molecular function category according to GO analysis results and involved the pathway of pentose and glucuronate interconversions, starch and sucrose metabolism, and steroid biosynthesis according to KEGG pathway analysis.

Most polysaccharides of *G. lingzhi* are miscellaneous polysaccharides, usually containing glucose and a small amount of arabinose, galactose, mannose, xylose, and other monosaccharides [37]. In the present study, DEGs of *G. lingzhi* isonuclear alloplasmic mainly involved the conversion of 1,4-α-D-galacturonide to glycerol in the pathway of pentose and glucuronate interconversions, which may affect the content of arabinose and galactose in polysaccharides. Remarkably, enriched starch and sucrose pathway metabolism included 1,3-β-Glucan and D-Glucose, which were considered to be the main chain of *G. lingzhi* polysaccharide [38]. In *G. lingzhi*, triterpenoids are synthesized via the mevalonate pathway (MVA). Farnesyl-PP to squalene and, finally, to lanosterol is an important part of MVA [26]. Enriched steroid biosynthesis pathway not only involved these metabolites, but also the downstream metabolic process to ergosterol. It well explains why mitochondria could affect the content of triterpenoids. Furthermore, the expression level of DEGs of the three pathways behaved differently in the other isonuclear alloplasmic strains, which proved the accuracy of the result. The three pathways were related to the synthesis and metabolism of the secondary metabolites in *G. lucidum*, polysaccharides and triterpenoid. This was roughly consistent with the previous results of content and reflected the influence of mitochondria on active components from the side. Although it is hard to explain how the mechanism of mitochondria affect the mycelial growth rate and basidiospore yield based on present transcriptome results, we believe that this work will contribute to a better understanding of mitochondria function and pave the way for screening of excellent strains and improving active components.

## 5. Conclusions

The present study illustrated that mitochondria may affect mycelial growth rate, basidiospore yield, and the content of polysaccharide and triterpenoid via structuring and comparing isonuclear alloplasmic strains. Moreover, based on the analysis of the transcriptome, pentose and glucuronate interconversions, starch and sucrose metabolism, and steroid biosynthesis play an important role in the variations in polysaccharide and triterpenoid content in isonuclear alloplasmic strains. This study will be a useful foundation for understanding of mitochondria and will be helpful for breeding improved varieties.

## Figures and Tables

**Table 1:** Mushroom yield, basidiospore yield, and polysaccharide and triterpenoid content in isonuclear alloplasmic strains S1–S5.

Strains	Mushroom Yield g/bag	Basidiospores Yield g/bag	Polysaccharide Content (mg/g)	Triterpenoid Content (mg/g)
S1	70.67 ± 3.42 ^a^	19.67 ± 1.34 ^a^	31.16 ± 0.97 ^a^	19.34 ± 0.38 ^d^
S2	70.67 ± 5.41 ^a^	15.94 ± 1.86 ^b^	29.19 ± 1.57 ^b^	20.04 ± 0.19 ^bc^
S3	70.83 ± 3.75 ^a^	16.89 ± 1.24 ^ab^	25.93 ± 0.85 ^d^	20.87 ± 0.50 ^b^
S4	73.17 ± 4.91 ^a^	15.33 ± 2.17 ^b^	26.58 ± 1.05 ^cd^	22.51 ± 0.76 ^a^
S5	66.67 ± 4.13 ^a^	5.55 ± 0.99 ^c^	28.00 ± 0.43 ^bc^	19.94 ± 0.26 ^c^

For polysaccharide and triterpenoid content: values are the mean ± standard deviation of three independent samples. For all other parameters: values are the mean ± standard deviation of 30 independent bag samples. Mean values in each column followed by lowercase indicate the difference among strains at the significance level of 0.05, and the same letters mean not significantly different (*p* > 0.05).

## Data Availability

All experimental data in this study will be made available upon reasonable request from readers.

## Supplementary Materials

The following supporting information can be downloaded at: https://www.mdpi.com/article/10.3390/jof8111182/s1, Figure S1: Pearson correlation analysis between samples among strain S1–S5; Table S1: Specific primers for nonconserved regions among nucleus DNA and mtDNA of *G. lucidum* strains; Table S2: Summary of RNA-Seq data.

## References

[1] Giordano L., Sillo F., Garbelotto M., Gonthier P. (2018). Mitonuclear interactions may contribute to fitness of fungal hybrids. Sci. Rep..

[2] Medina R., Franco M.E.E., Bartel L.C., Alcántara V.M., Saparrat M.C.N., Balatti P.A. (2020). Fungal mitogenomes: Relevant features to planning plant disease management. Front. Microbiol..

[3] Kouvelis V.N., Hausner G. (2022). Editorial: Mitochondrial genomes and mitochondrion related gene insights to fungal evolution. Front. Microbiol..

[4] Al-Reedy R.M., Malireddy R., Dillman C.B., Kennell J.C. (2012). Comparative analysis of *Fusarium* mitochondrial genomes reveals a highly variable region that encodes an exceptionally large open reading frame. Fungal Genet. Biol..

[5] Freel K.C., Friedrich A., Schacherer J. (2015). Mitochondrial genome evolution in yeasts: An all-encompassing view. FEMS Yeast Res..

[6] Liu W., Cai Y., Zhang Q., Shu F., Chen L., Ma X., Bian Y.B. (2020). Subchromosome-scale nuclear and complete mitochondrial genome characteristics of *Morchella crassipes*. Int. J. Mol. Sci..

[7] O’Connor E., McGowan J., McCarthy C., Amini A., Grogan H., Fitzpatrick D.A. (2019). Whole genome sequence of the commercially relevant mushroom strain *Agaricus bisporus* var. bisporus ARP23. G3 Genes Genomes Genet..

[8] Liu X.R., Wu X.P., Tan H., Xie B.G., Deng Y.J. (2020). Large inverted repeats identified by intra-specific comparison of mitochondrial genomes provide insights into the evolution of *Agrocybe aegerita*. Comput. Struct. Biotechnol. J..

[9] Bashir K.M.I., Rheu K.M., Kim M.S., Cho M.G. (2020). The complete mitochondrial genome of an edible mushroom, *Sparassis crispa*. Mitochondrial DNA Part B Resour..

[10] Song Y., Wan J.N., Shang J.J., Feng Z., Jin Y., Li H., Guo T., Wu Y.Y., Bao D.P., Zhang M. (2022). The complete mitochondrial genome of the edible mushroom *Grifola frondosa*. Mitochondrial DNA Part B Resour..

[11] Li J.Q., Zhang J.H., Chen H.M., Chen X.D., Lan J., Liu C. (2013). Complete mitochondrial genome of the medicinal mushroom *Ganoderma lucidum*. PLoS ONE.

[12] Li Q., Xiang D.B., Wan Y., Wu Q., Wu X.Y., Ma C.R., Song Y., Zhao G., Huang W.L. (2019). The complete mitochondrial genomes of five important medicinal *Ganoderma* species: Features, evolution, and phylogeny. Int. J. Biol. Macromol..

[13] Steensels J., Gallone B., Verstrepen K.J. (2021). Interspecific hybridization as a driver of fungal evolution and adaptation. Nat. Rev. Microbiol..

[14] Moon S.K., Thompson L.J., Madamanchi N., Ballinger S., Papaconstantinou J., Horaist C., Runge M.S., Patterson C. (2001). Aging, oxidative responses, and proliferative capacity in cultured mouse aortic smooth muscle cells. Am. J. Physiol. Heart Circ. Physiol..

[15] Latorre-Pellicer A., Moreno-Loshuertos R., Lechuga-Vieco A.V., Sánchez-Cabo F., Torroja C., Acín-Pérez R., Calvo E., Aix E., González-Guerra A., Logan A. (2016). Mitochondrial and nuclear DNA matching shapes metabolism and healthy ageing. Nature.

[16] Sun X.M., Wang H.H., Han X.F., Chen S.W., Zhu S., Dai J. (2014). Fingerprint analysis of polysaccharides from different *Ganoderma* by HPLC combined with chemometrics methods. Carbohydr. Polym..

[17] Qu Z.W., Zhou S.Y., Guan S.X., Gao R., Duan Z.W., Zhang X., Sun W.Y., Fan W.L., Chen S.S., Chen L.J. (2018). Recombinant expression and bioactivity comparison of four typical fungal immunomodulatory proteins from three main *Ganoderma* Species. BMC Biotechnol..

[18] Liu Y.C., Tang X.C., Hu H.P., Chen D.L., Xie Y.Z., Liang X.W., Li X.M., Xiao C., Huang L.H., Wu Q.P. (2021). Genetic diversity and main functional composition of *Lingzhi* strains from main producing areas in China. AMB Express..

[19] Xu J.W., Zhao W., Zhong J.J. (2010). Biotechnological production and application of ganoderic acids. Appl. Microbiol. Biotechnol..

[20] Gong X., Ji M.Y., Xu J.P., Zhang C.H., Li M.H. (2020). Hypoglycemic effects of bioactive ingredients from medicine food homology and medicinal health food species used in China. Crit. Rev. Food Sci. Nutr..

[21] He X.R., Fang J.C., Guo Q., Wang M., Li Y.S., Meng Y.B., Huang L.H. (2020). Advances in antiviral polysaccharides derived from edible and medicinal plants and mushrooms. Carbohydr. Polym..

[22] Hu Y., Wang S.X., Wu F.Y., Wu K.J., Shi R.P., Qin L.H., Lu C.F., Wang S.Q., Wang F.F., Zhou S.B. (2022). Effects and mechanism of *Ganoderma lucidum* polysaccharides in the treatment of diabetic nephropathy in streptozotocin-induced diabetic rats. BioMed Res. Int..

[23] Chen S.D., Guan X.Y., Yong T.Q., Gao X., Xiao C., Xie Y.Z., Chen D.L., Hu H.P., Wu Q.P. (2022). Structural characterization and hepatoprotective activity of an acidic polysaccharide from *Ganoderma lucidum*. Food Chem. X.

[24] Ye L.Y., Deng Y.J., Mukhtar I., Meng G.L., Song Y.J., Cheng B., Hao J.B., Wu X.P. (2020). Mitochondrial genome and diverse inheritance patterns in *Pleurotus pulmonarius*. J. Microbiol..

[25] Chen B.Z., Ke B.R., Ye L.Y., Jin S.S., Jie F., Zhao L.L., Wu X.P. (2017). Isolation and varietal characterization of *Ganoderma resinaceum* from areas of *Ganoderma lucidum* production in China. Sci. Hortic..

[26] Ye L.Y., Liu S.R., Xie F., Zhao L.L., Wu X.P. (2018). Enhanced production of polysaccharides and triterpenoids in *Ganoderma lucidum* fruit bodies on induction with signal transduction during the fruiting stage. PLoS ONE.

[27] Pertea M., Kim D., Pertea G.M., Leek J.T., Salzberg S.L. (2016). Transcript-level expression analysis of RNA-seq experiments with HISAT, StringTie and Ballgown. Nat. Protoc..

[28] Hao J.B., Ye L.Y., Meng G.L., Song Y.J., Fu J.S., Wu X.P. (2021). The protective effect and crucial biological pathways analysis of *Trametes lactinea* mycelium polysaccharides on acute alcoholic liver injury in mice based on transcriptomics and metabolomics. Food Sci. Hum. Well..

[29] Young M.D., Wakefield M.J., Smyth G.K., Oshlack A. (2010). Gene ontology analysis for RNA-seq: Accounting for selection bias. Genome Biol..

[30] Wei Z.H., Liu L.L., Guo X.F., Li Y.J., Hou B.C., Fan Q.L., Wang K.X., Luo Y.D., Zhong J.J. (2016). Sucrose fed-batch strategy enhanced biomass, polysaccharide, and ganoderic acids production in fermentation of *Ganoderma lucidum* 5.26. Bioprocess Biosyst. Eng..

[31] Tang Y.J., Zhu L.W. (2010). Improvement of ganoderic acid and *Ganoderma* polysaccharide biosynthesis by *Ganoderma lucidum* fermentation under the inducement of Cu^2+^. Biotechnol. Prog..

[32] Sun B., You H., Xu J.W. (2021). Enhancement of ganoderic acid production by promoting sporulation in a liquid static culture of *Ganoderma* species. J. Biotechnol..

[33] Ma Y.H., Zhang Q.Q., Zhang Q.F., He H.Q., Chen Z., Zhao Y., Wei D., Kong M.G., Huang Q. (2018). Improved production of polysaccharides in *Ganoderma lingzhi* mycelia by plasma mutagenesis and rapid screening of mutated strains through infrared spectroscopy. PLoS ONE.

[34] Shi D.K., Zhu J., Sun Z.H., Zhang G., Liu R., Zhang T.J., Wang S.L., Ren A., Zhao M.W. (2017). Alternative oxidase impacts ganoderic acid biosynthesis by regulating intracellular ROS levels in *Ganoderma lucidum*. Microbiology.

[35] Mezhnina V., Ebeigbe O.P., Poe A., Kondratov R.V. (2022). Circadian control of mitochondria in reactive oxygen species homeostasis. Antioxid. Redox Signal..

[36] Suski J., Lebiedzinska M., Bonora M., Pinton P., Duszynski J., Wieckowski M.R. (2018). Relation between mitochondrial membrane potential and ROS formation. Methods Mol. Biol..

[37] Miyazaki T., Nishijima M. (1981). Studies on fungal polysaccharides. XXVII. Structural examination of a water-soluble, antitumor polysaccharide of *Ganoderma lucidum*. Chem. Pharm. Bull..

[38] Liu G., Zhang J., Kan Q., Song M., Hou T., An S., Lin H., Chen H., Hu L., Xiao J. (2022). Extraction, structural characterization, and immunomodulatory activity of a high molecular weight polysaccharide from *Ganoderma lucidum*. Front. Nutr..

