# Supplementary material for: The Effect of Mitochondria on Ganoderma lucidum Growth and Bioactive Components Based on Transcriptomics
Source: J Fungi (Basel). 2022 Nov 9;8(11):1182. doi: 10.3390/jof8111182 (PMC9692720; doi:10.3390/jof8111182)

# The Effect of Mitochondria on *Ganoderma lucidum* Growth and Bioactive Components Based on Transcriptomics

**Liyun Ye [1], Xiaofang He [1], Congbao Su [1], Haiying Feng [1], Guoliang Meng [1], Bingzhi Chen [2,3] and Xiaoping Wu [1,*]**

[1] College of Life Sciences, Fujian Agriculture and Forestry University, Fuzhou 350002, China
[2] College of Food Sciences, Fujian Agriculture and Forestry University, Fuzhou 350002, China
[3] Key Laboratory of Subtropical Characteristic Fruits, Vegetables and Edible Fungi Processing (Co-Construction by Ministry and Province), Ministry of Agriculture and Rural Affairs, Fuzhou 350002, China
* Correspondence: fjwxp@126.com

## Title page

**Figure 1. The process of constructing isonuclear alloplasmic.**

**Figure 2. The PCR results of isonuclear alloplasmic strain S1-S5.** (A) The PCR results of samples strain by the primers of nuclear genome. M indicates the 2,000 bp DNA marker; lane 1-5, 6-10, 11-15, 16-20, and 21-25 template DNA was S1-S5 in each group and primer were Nc-119, Nc-TL, Nc-WBL, Nc-SL4, and Nc-SL10, separately; lane 26 is negative control with $ddH_2O$ instead of DNA. (B) The PCR results of samples strain by the primers of mtDNA. M indicates the 2,000 bp DNA marker; lane 1-4 template DNA was S1and primer were primer 6-9 in table s1; lane 5-8 template DNA was S2 and primer were primer 6-9 in table s1; lane 9-12 template DNA was S3 and primer were primer 6-9 in table s1; lane 13-16 template DNA was S4 and primer were primer 6-9 in table s1; lane 17-20 template DNA was S5 and primer were primer 6-9 in table s1; lane 21 is negative control with $ddH_2O$ instead of DNA.

**Figure 3. Biological characteristics of five isonuclear alloplasmic strains.** (A). colony and fruit bodies morphology. (B) mycelial growth rate in PDA. (C) mycelal growth rate in solid-state fermentation. The same letters mean not significantly different ($p>0.05$).

**Figure 4. Correlation and DEGs analysis between strain S1 and S5.** (A) Pearson correlation analysis between samples of strain S1 and S5. (B) DEGs between strain S1 and S5.

**Figure 5 Enrichment analysis of DEGs.** (A) GO enrichment of DEGs. (B) Bubble plot of KEGG enrichment of DEGs.

**Figure 6. Polysaccharide and triterpenoid related pathways of isonuclear alloplasmic based on transcriptome analysis.** (A) pentose and glucuronate interconversions pathway. (B) starch and sucrose metabolism pathway. (C) steroid biosynthesis pathway; (D) Changes in expression of genes involved in pathways.

**Table 1.** Mushroom yield, basidiospores yield, and polysaccharide and triterpenoid

content in isonuclear alloplasmic strains S1-S5.

**Table S1.** Specific primers for nonconserved regions among nucleus DNA and mtDNA of *G.lucidum* strains.
Table S2. Summary of RNA-Seq data.

**Figure S1.** Pearson correlation analysis between samples among strain S1-S5.

## Supplementary tables and figures

Table S1. Specific primers for nonconserved regions among nucleus DNA and mtDNA of *G.lucidum* strains.

| Primer number | Primer name | Strain | Location | Primer sequence (5‘→3’) | Production size (bp) |
|---|---|---|---|---|---|
| 1 | Nc-119 | M119-M71 | nucleus DNA | F:GACAACCAGTCACGCTCAAG;<br>R:CTATCCCATCCGTACTCCTG | 418 |
| 2 | Nc-TL | MTL | nucleus DNA | F:TTCACATCCCTCTCGTCTATGC;<br>R:TACTCCCCTCCTGATCTTTCCT | 643 |
| 3 | Nc-WBL | MWBL | nucleus DNA | F:CGCAACACACAGGAGAATCAAC;<br>R:CAACTCAGACTTTCC | 594 |
| 4 | Nc-SL10 | MSL10 | nucleus DNA | F:GTACATCCGTTCGGCTTCG;<br>R:TGCTTTGGTGGGGTTCAAG | 449 |
| 5 | Nc-SL4 | MSL4 | nucleus DNA | F:ACCAAAACCATGAAATCCAACAG;<br>R:GGAGGGAGATAATCAAACGAAAA | 465 |
| 6 | Mito-119-TL | MTL; M119-M71 | mtDNA | F:TAGCGGTCTTAACTATGAGGA;<br>R:GGTATTCCATCTTTCGTCAAT | MTL:261;<br>M119-M71:1933 |
| 7 | Mito-119-WBL | MWBL;<br>M119-M71 | mtDNA | F:AACCATATCTCACACACCATCT;<br>R:ATCAGCACTTTTTTGTAATTCC | MWBL:1117;<br>M119-M71:824 |
| 8 | Mito-119-SL10 | MSL10;<br>M119-M71 | mtDNA | F:GATAGGTACTGCATTCTTGGG;<br>R:CAGGAACAATAGATGCTGGTG | MSL10:1491;<br>M119-M71:401 |
| 9 | Mito-119-SL4 | MSL4;<br>M119-M71 | mtDNA | F:TATCAGCACCTGGAGTGCAAGT;<br>R:CATCTCCTTCAATTAACCCTGC | MSL4:253;<br>M119-M71:1528 |

Table S2. Summary of RNA-Seq data.

| Sample | Raw Reads | Raw Bases | Clean Reads | Clean Bases | Error Rate | Q20 | Q30 | GC Content |
|---|---|---|---|---|---|---|---|---|
| S1_1 | 21559906 | 3.23G | 21464430 | 3.22G | 0.03% | 97.60% | 93.68% | 59.67% |
| S1_2 | 25145530 | 3.77G | 25033436 | 3.76G | 0.03% | 97.54% | 93.54% | 59.56% |
| S1_3 | 22296198 | 3.34G | 22206522 | 3.33G | 0.03% | 97.56% | 93.60% | 59.70% |
| S2_1 | 27409858 | 4.11G | 27312980 | 4.1G | 0.03% | 97.72% | 93.95% | 59.62% |
| S2_2 | 30734054 | 4.61G | 30620530 | 4.59G | 0.03% | 97.55% | 93.63% | 59.52% |
| S2_3 | 22500770 | 3.37G | 22421140 | 3.36G | 0.03% | 97.72% | 93.90% | 59.41% |
| S3_1 | 24568268 | 3.68G | 24468368 | 3.67G | 0.03% | 97.72% | 93.91% | 59.53% |
| S3_2 | 23816140 | 3.57G | 23720756 | 3.56G | 0.03% | 97.65% | 93.84% | 59.55% |
| S3_3 | 20727876 | 3.1G | 20650116 | 3.1G | 0.03% | 97.80% | 94.09% | 59.50% |
| S4_1 | 22344306 | 3.35G | 22264820 | 3.34G | 0.03% | 97.79% | 94.00% | 59.66% |
| S4_2 | 30760148 | 4.61G | 30650670 | 4.6G | 0.03% | 97.79% | 94.08% | 59.63% |
| S4_3 | 26272338 | 3.94G | 26165408 | 3.92G | 0.03% | 97.51% | 93.63% | 59.60% |
| S5_1 | 28972968 | 4.34G | 28860520 | 4.33G | 0.03% | 97.53% | 93.64% | 59.70% |
| S5_2 | 19012984 | 2.85G | 18930552 | 2.84G | 0.03% | 97.61% | 93.68% | 59.50% |
| S5_3 | 24246148 | 3.63G | 24147638 | 3.62G | 0.03% | 97.55% | 93.67% | 59.61% |

Figure S1. Pearson correlation analysis between samples among strain S1-S5.

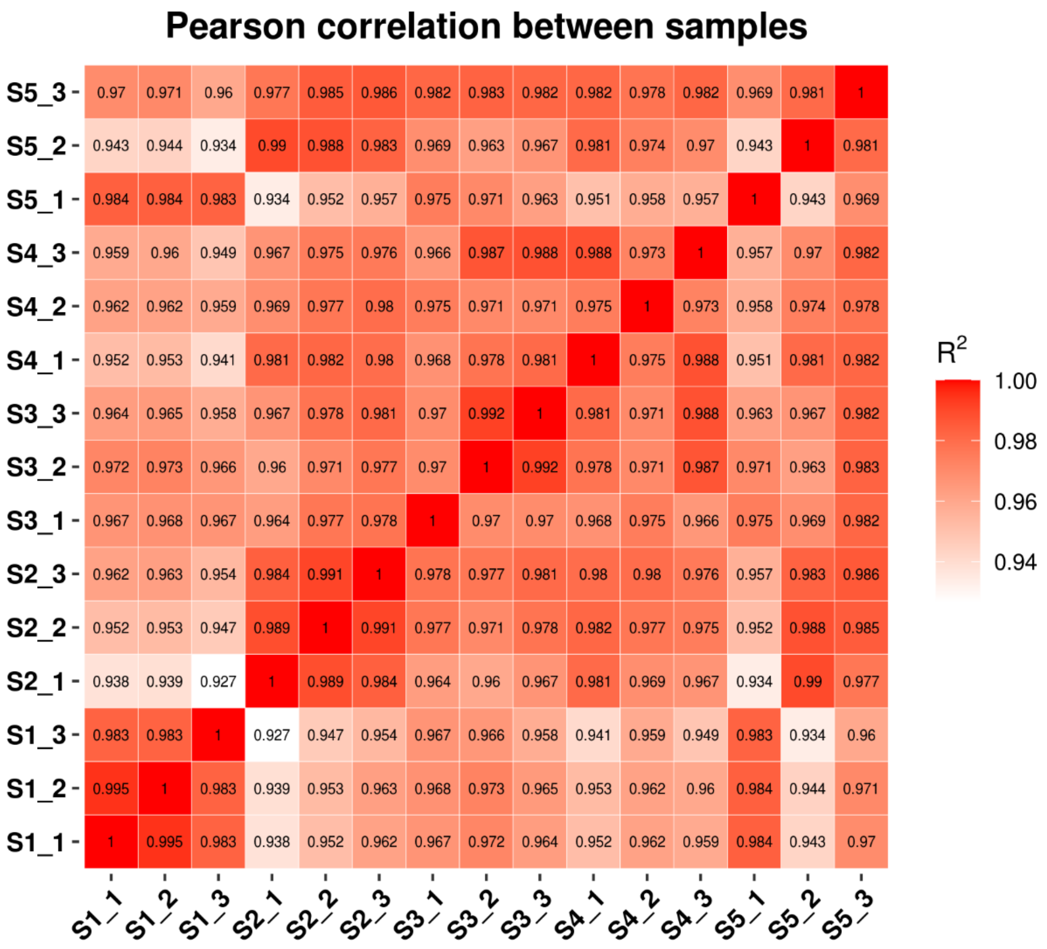

Supplement: Supplementary file 1 [file jof-08-01182-s001.zip › jof-1965920-supplementary.pdf]
